# The Statisics of Cretinism

**Published:** 1850-03

**Authors:** 


The Statisics of Cretinism.—" Amongst the 2,188,000 souls, forming the population of Confederate Switzerland, there are about 20,000 persons afflicted with cretinism in a greater or less degree; about 8000 of these, it is calculated, are truly idiots; while the others labor under various kinds of bodily and mental infirmities, sufficient to mark the existence of the same malady, but not to prevent them from engaging in the ordinary occupations of life.—*Ranking's Abstract.*

